# Role of the rostral anterior cingulate cortex in emotion processing in Treatment Resistant Depression

**DOI:** 10.1038/s41398-025-03600-3

**Published:** 2025-10-06

**Authors:** Ana Rita Barreiros, Isabella Breukelaar, Prashanth Mayur, Jagadeesh Andepalli, Yoshiro Tomimatsu, Kenta Funayama, Sheryl Foster, Anthony Harris, Mayuresh Korgaonkar

**Affiliations:** 1 Brain Dynamics Centre, Westmead Institute for Medical Research, Sydney, NSW Australia; 2 Westmead Clinical School, Faculty of Medicine and Health, University of Sydney, Sydney, NSW Australia; 3 Black Dog Institute, Sydney, NSW Australia; 4 School of Clinical Medicine, Faculty of Medicine and Health, University of New South Wales, Sydney, NSW Australia; 5 Mood Disorders Unit, Cumberland Hospital, Western Sydney Local Health District, Parramatta, NSW Australia; 6 Takeda Neuroscience Therapeutic Area Unit, Cambridge, MA USA; 7 Research, Takeda Pharmaceutical Company Ltd., Kanagawa Fujisawa, Japan; 8 Department of Radiology, Westmead Hospital, Westmead, NSW Australia; 9 School of Health Sciences, Faculty of Medicine and Health, The University of Sydney, Sydney, NSW Australia; 10 Specialty of Psychiatry, Sydney Medical School, Faculty of Medicine and Health, The University of Sydney, Sydney, NSW Australia

**Keywords:** Depression, Molecular neuroscience

## Abstract

The rostral anterior cingulate cortex (rACC) has been identified as a key region in treatment-resistant depression (TRD), potentially influencing the adaptive interplay between the default mode network and other critical neural networks. This study aims to further uncover the role of the rACC in TRD by investigating its differential connectivity during tasks that evoke conscious and non-conscious emotional responses. Thirty-one TRD patients, 35 treatment-sensitive depression (TSD) patients, and 37 healthy controls (HC) underwent 3T fMRI while performing tasks designed to elicit emotional responses to facial expressions under supraliminal and subliminal conditions. Connectivity patterns of the rACC were analyzed using seed-based and region-of-interest (ROI) approaches. During the processing of positive emotions in the subliminal task, TRD patients showed *increased* rACC connectivity to the cerebellum and middle temporal gyrus, compared to HC and TSD. Furthermore, significant *hypoconnectivity* between the rACC and hippocampus was found in the TRD, during the supraliminal processing of positive emotions, compared to TSD and HC. Altered neural connectivity to both subliminal and supraliminal processing of positive facial emotion distinguishes TRD from healthy individuals and patients who respond to depression treatments. This observation fits with anhedonia symptoms that persist in TRD and our findings point to altered connectivity between the rACC and regions involved in emotion regulation and contextualisation during non-conscious processing of positive stimuli.

## Introduction

Treatment-resistant depression (TRD) represents a significant clinical challenge, characterized by a persistent lack of response to standard antidepressant treatments. This condition profoundly affects patients’ quality of life and stresses the need for a deeper exploration into the complex brain dynamics that contribute to its persistence.

The investigation of emotion processing in TRD is particularly crucial given its central role in depressive symptomatology [1]. Emotion processing deficits, characterized by impairments in recognizing, interpreting, and responding to emotional stimuli, are a hallmark of major depressive disorder (MDD) [2–5], including its treatment-resistant forms [1]. Studies have shown that patients with TRD often exhibit altered neural responses to emotional stimuli, reflecting dysregulation in key brain areas and networks involved in emotion processing. Some of these findings have emerged from studies examining neural changes following ketamine treatment, highlighting the modulation of emotion-related circuits in TRD [6–8].

The understanding of the pathophysiology of depression has shifted to a model based on dysregulation of neural networks, rather than a single neuroanatomical location [9]. Central to these investigations are neural networks like the default-mode network (DMN), which is known for its role in self-referential thought processes and mind-wandering. The DMN exhibits peak activity during periods of restful introspection and is diminished when engaging in tasks that require external goal-oriented focus [10]. This reduction in activity, or ‘deactivation,’ is believed to indicate a shift from internal processing to increased attention on external tasks [10, 11].

The rostral anterior cingulate cortex (rACC), a key node within the DMN, is intricately linked with regions and networks vital for emotional and cognitive regulation [12, 13]. It plays a role in transitions between internally and externally focused cognitive states and may modulate connectivity between the DMN and other networks during emotional and attentional processing [14–17].

rACC dysfunction has been associated with features of depression such as negative thinking, attentional bias, and poor emotion regulation [18–20]. At rest, lower rACC activity has been reported in patients who do not respond to treatments, compared to responders across modalities including pharmacotherapy [21], sleep intervention [22], and rTMS [13]. In contrast, some studies suggest that heightened rACC activity may underlie persistent rumination and may appear in specific subtypes or task contexts [23–26], highlighting the complexity of this region’s role.

Moreover, rACC activity may reflect the degree of treatment resistance [27]. Our previous work demonstrated that patients unresponsive to advanced treatments like rTMS and ECT showed greater rACC activity at rest compared to those treated with first-line interventions (psychotherapy or pharmacotherapy) [28]. This finding suggests that resting-state rACC activity could vary depending on treatment stage or resistance level. However, a significant gap remains in our understanding of how these neural networks function during active emotional processing tasks. While resting-state fMRI has revealed much about the role of rACC in TRD, task-based fMRI can provide additional insights into the interplay of the rACC and the key brain regions that are activated and modulated in response to emotional stimuli. This approach is particularly relevant for understanding the neural basis of emotional dysregulation in TRD.

Emotional processing occurs at multiple levels of awareness, encompassing both conscious (supraliminal) and non-conscious (subliminal) perception. Subliminal processing refers to the perception of emotional stimuli below the threshold of conscious awareness, often eliciting automatic and implicit emotional responses [29]. In contrast, supraliminal processing involves the conscious recognition and appraisal of emotional information [29]. Investigating both levels is crucial, as they may engage different neural circuits and contribute distinctively to emotional regulation and dysregulation in mood disorders [30]. Prior research suggests that subliminal emotional cues can influence affective biases and automatic negative thoughts, which are central features of depression, while supraliminal processing relates more directly to conscious emotional experience and rumination [29]. Understanding these differential processes, particularly how they modulate connectivity within key brain networks such as the DMN, may offer important insights into the mechanisms underlying treatment resistance in depression. Examining these processes in parallel allows us to test whether connectivity alterations in TRD reflect disruptions in automatic emotion reactivity, higher-order appraisal, or both. By assessing connectivity rather than just activation, we also aim to capture the dynamic interplay between the rACC and other affective or regulatory regions that may mediate these effects.

Therefore, the goal of our investigation was to extend our understanding of the neural underpinnings of TRD, particularly the role of the rACC beyond resting-state conditions and provide a more comprehensive view of the involvement of this region in the disorder’s complex neurobiology. Specifically, this study evaluated the functional connectivity of the rACC in treatment-resistant depression, during supraliminal and subliminal level of processing of both negative and positive emotions. Our study included both healthy individuals and treatment responsive patients with depression for comparison.

## Materials and methods

### Participants

Initially, 39 individuals with TRD and 35 individuals with TSD were recruited through a local network of specialist psychiatrists and clinics. TRD and TSD individuals met DSM-5 criteria for primary diagnosis of MDD, assessed through the Structured Clinical Interview for the DSM-5 (SCID-5) [31]. The inclusion criteria for TRD were: no remission of symptoms with at least two adequate trials (in terms of dosage, duration – 6 weeks for each trial) of antidepressant of different pharmacologic classes, as well as the presence of moderate to severe depressive symptoms (assessed by a rating greater or equal to 16 in the 21-item Hamilton Depression Rating Scale – HAMD-21 [32]. Participants in the TSD group were identified as patients who had achieved complete remission of symptoms for at least two weeks (characterized by a HAMD-21 score of less than or equal to 9). The control group (HC) comprised of 38 healthy individuals recruited through community advertisements with no psychiatric illnesses, assessed using the SCID-5. All participants were aged between 18 and 65 years old.

For both patient groups, indices of illness severity and chronicity were assessed. These indices included age of onset, number of inpatient hospitalizations, length of remission period since last episode, severity of worst episode (Clinical Global Impression – BP, CGI-BP [33]. number of previous depressive episodes, history of suicidal ideation and behaviour, and history of suicide attempt. Information on past and current medication and other forms of treatment (e.g. ECT, or TMS) was also collected. Level of functioning was assessed by the Social and Occupational Functioning Assessment Scale (SOFAS) [34].

HC and patient groups (TRD and TSD) were matched for age, sex and education status. Exclusion criteria for all participants included (a) inability to provide consent, (b) insufficient English proficiency, (c) current primary diagnosis of eating disorder, psychosis, personality disorder or primary PTSD, (d) substance dependence for the past 3 months, (e) pregnancy, (f) history or current neurological disorder or prior brain injury, (g) ECT or TMS in the last 6 months, (h) contraindication to MRI.

Data collection was conducted at Westmead Hospital, Department of Radiology and at the Brain Dynamics Centre, The Westmead Institute for Medical Research, in Sydney, Australia. The research protocol was approved by the Western Sydney Local Health District Human Research Ethics Committee and all participants provided written consent.

### MRI data collection

Functional imaging was performed on a Siemens Prisma 3T MRI system running VE11C software and utilising a 2D GRE EPI sequence with a 64-channel head/neck array coil. Whole brain data for both faces tasks was acquired in the axial oblique plane with 47 interleaved slices of 3 mm thickness/0 mm gap parallel to the AC-PC line. Other parameters were as follows: TR/TE = 2500 ms/27 ms, Flip angle = 90°, FOV = 240 mm, matrix = 86 × 86, voxel size = 3 mm × 2.8 mm × 2.8 mm, parallel imaging r = 2, number of volumes = 120. Total acquisition time was 5 min 13 s.

### Task description

The emotion processing tasks utilized in this study were based on established paradigms previously described in [29, 35]. These tasks involve the presentation of facial expressions categorized into various emotional states: threat-related emotions (fear and anger), disgust, loss-related emotions (sadness), reward-related emotions (happiness), and neutral expressions. The stimuli are derived from a standardized series of facial expressions and are modified for central positioning at eye level.

There were two tasks: supraliminal emotional face processing, and subliminal emotional face processing, each analysed separately.

In the subliminal task, each emotional face is briefly presented for 16.7 ms, followed by a perceptual mask of a neutral face for 150 ms and an interstimulus interval of 1233.3 ms. This presentation is crafted to be below the level of conscious awareness. Behavioral psychophysical testing, as detailed in Williams et al. [29], confirms that this duration is sufficient to meet the criteria for subliminal threshold detection.

The supraliminal task differs in presentation duration, with each emotional face displayed for 500 ms, based on evidence that this duration ensures conscious processing of the emotion stimulus [29]. The interstimulus interval in this condition is 750 ms. For both subliminal and supraliminal task presentations, faces are grouped in blocks by emotion, with each block containing eight faces and repeated five times, totalling 240 stimuli.

Throughout both paradigms, participants are instructed to focus on each face, with no specific behavioral responses required during scanning. This approach is consistent with findings that passive processing of emotional stimuli can elicit significant brain activation [30].

### MRI data processing

MRI data was processed and analysed using Matlab R2018b (The Mathworks inc, Natick, Massachusetts), SPM12 (Wellcome Trust Centre for Neuroimaging, London, UK), CONN functional connectivity toolbox v16b. Details of pre-processing steps are presented in our previous study [36]. Four TRD participant datasets were excluded for excessive head motion during the scan. Five participants who did not complete the Faces tasks were identified and subsequently excluded from the analysis. This resulted in 31 TRD, 35 TSD and 37 healthy controls for final analyses.

### Demographic and clinical characteristics

All three groups were compared for age and gender (demographic variables), using a one-way ANOVA and chi square test, respectively. The TRD and TSD groups were compared for age of onset, age of first episode, depression severity (HAMD-21 score), functionality (SOFAS score), severity of worst depressive episode, number of previous depressive episodes, using student t-tests. The groups were also compared for history of hospitalizations, suicidal attempts and suicidal ideation, using chi square tests of independence.

### Functional connectivity (FC) analyses

Generalized PsychoPhysiological Interaction (gPPI) analyses, as employed in this study, are designed to explore task-specific changes in the BOLD signal across different brain regions over time. The gPPI approach is particularly suited for studies involving multiple task conditions, such as the varied emotional stimuli in our tasks.

In this analysis, the interaction term is created by convolving each task condition’s onset times, representing different emotions, with the hemodynamic response function. This term is then multiplied by the seed region’s estimated neural activity, derived from the deconvolved BOLD signal. The gPPI model is distinct from standard PPI in that it includes the interaction factors from all conditions simultaneously in the estimation model in order to better account for between-condition overlaps. In this analysis, PPI effects (interaction terms in PPI model) are relative to the baseline state (the baseline state is defined by the zero values of the interaction term), so they provide a relative measure of connectivity characterizing differential task-specific effects.

One critical contrast employed was “Happy” versus “Neutral,” where “Neutral” represents emotionally neutral faces. This contrast was chosen to elucidate the neural mechanisms underlying positive emotion processing. By comparing brain activity associated with viewing happy faces against neutral faces, we aimed to identify the specific brain regions and their interconnections that are selectively engaged in the perception and processing of positive emotional stimuli.

Additionally, we analyzed the aggregated negative valenced conditions (“Sadness”, “Anger”, “Fear”, and “Disgust”) versus “Neutral” to investigate the networks involved in negative emotion processing. By averaging together these negative conditions and contrasting them with neutral emotion processing, we aimed to capture a broad perspective of the brain’s response to negative stimuli.

In this study, the primary gPPI analysis employed a seed-to-voxel whole brain approach to comprehensively examine the role of the rACC in emotional processing in this population.

The selection of rACC ROI anatomic landmark derived from the voxels reported by Pizzagalli and colleagues [37], and also used in our prior studies on rACC [28]. The functional rACC ROI was a unilateral 10 × 10 × 10 mm cube placed around the central MNI coordinate for the left rACC ((−10 45 −5). FC values were calculated from the rACC voxel-wise to the rest of the brain for each participant in the dataset as bivariate Fisher’s z-transformed correlation coefficients. This measures the association between the rACC seed BOLD timeseries and each voxel of the whole brain BOLD timeseries using generalized linear model (GLM).

The statistical parametric maps threshold was set *p* < 0.05 at the cluster-level family-wise error (FWE)-corrected for multiple comparisons, using an initial whole-brain voxel-wise *p* < 0.001. We used an arbitrary threshold of a minimum cluster size of 20 as necessary to be considered relevant.

### Clinical factors

In order to analyse the associations between the FC measures and treatment resistance in the TRD group, we computed a variable to reflect chronicity of the disorder, by calculating the number of years from age of onset to current age, which is referred to as “number of years since onset”. The relationships between the mean FC values for the significant ROIs and different clinical factors (depression severity, functionality, severity of worst depressive episode, number of previous depressive episodes, and number of years since onset) in the two patient groups separately were analysed using Pearson correlation coefficients.

All effects considered significant at the *p* < 0.05 significance level corrected for multiple comparisons, using a Bonferroni adjustment. Statistical analyses were performed using SPSS software version 21 (IBM Corp, 2012).

## Results

### Demographic and clinical characteristics

Demographic and clinical data for the final sample are summarized in Table 1. All three groups were comparable for age and sex. As expected, groups were significantly different in some clinical variables, reflecting a more severe clinical profile for TRD patients. Although the depression groups weren’t different for the number of past depressive episodes, the TRD group had higher rates of history of hospitalizations, history of ECT, and history of suicidal attempts.

**Table 1:** Summary of demographic and clinical characteristics of the sample.

	TRD (32)	TSD (34)	HC (37)	F/t/X^2	sig
*Demographics*					
Age, Mean ± SD [Min-Max]	42.4 ± 13.8 [18.1–64.3]	37.4 ± 11.1 [20.0–57.4]	42.29 ± 14.1 [18.9–65.9]	n.s.	n.s.
Gender (M), N (%)	12 (38)	17 (50)	17 (44.7)	n.s.	n.s.
*Clinical Profile*					
Age of onset, Mean ± SD [Min-Max]	29.97 ± 13.52 [8–53]	21.68 ± 9.40 [8–50]	n.a	n.s.	n.s.
Number of previous MDE, Mean ± SD [Min-Max]	8 ± 11 [1–40]	6.11 ± 6.16 [1–30]	n.a	n.s.	n.s.
Severity of worst MDE, Mean ± SD [Min-Max]	6.56 ± 1.13 [5–7]	5.52 ± 1.28 [3–7]	n.a	3.318	0.002
HAM-D-21 score, Mean ± SD [Min-Max]	23.66 ± 6.62 [15–39]	3.9 ± 2.92 [0–9]	n.a	15.611	0.000
SOFAS score, Mean ± SD [Min-Max]	74.45 ± 15.98 [40–100]	89.59 ± 5.56 [78–95]	n.a	−5.052	0.000
History of Hospitalizations, N (%)	22 (68)	6 (17.6)	n.a	17.625	0.000
History of ECT, N (%)	8 (25)	0 (0)	n.a	9.672	0.002
History of TMS, N (%)	2 (6.3)	0 (0)	n.a	n.a.	n.a.
History of Suicidal Ideation, N (%)	25 (78)	26 (76.5)	n.a	n.s.	n.s.
History of Suicidal Attempt, N (%)	17 (53)	5 (14.7)	n.a	12.439	0.000
**Current medications**					
SSRIs, N (%)	6 (18.8)	12 (35.3)	n.a.	n.s.	n.s.
SNRIs, N (%)	6 (18.8)	7 (20.6)	n.a.	n.s.	n.s.
Other ADM, N (%)	7 (21.9)	4 (11.8)	n.a.	n.s.	n.s.
Mood stabilizer, N (%)	1 (3.1)	2 (5.9)	n.a.	n.s.	n.s.
Anticonvulsants, N (%)	5 (15.6)	0 (0)	n.a.	6.282	0.012
Antipsychotics, N (%)	7 (21.9)	3 (8.8.)	n.a.	n.s.	n.s.

*n.a*. not applicable, *n.s*. not significant, *SD* standard deviation, *M* male, *MDE* major depressive episode, *HAMD-21* hamilton depression rating scale, 21 items, *SOFAS* social and occupational functioning assessment scale, *CGI-S* clinical global impression, severity, *ECT* electroconvulsive therapy, *TMS* transcranial magnetic stimulation, *N* total number, *SSRI* selective serotonin reuptake inhibitors, *SNRI* serotonin-norepinephrine reuptake inhibitors, *ADM* antidepressant medication.

There were no significant differences between groups for motion during the scan, after the exclusion of the four TRD participants for excessive motion.

### Whole brain seed-based connectivity analyses – rACC

#### Supraliminal processing

**Fig. 1:**
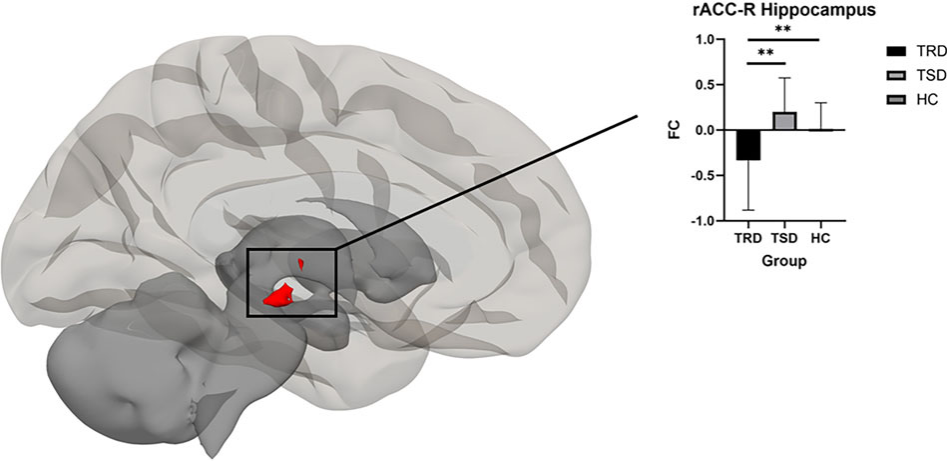
Differences between the groups for the rACC functional connectivity during supraliminal processing of emotional stimuli. This illustration offers a visualization of the cluster that contribute to differences between the three groups (Treatment-Resistant Depression (TRD) and Treatment-Sensitive Depression (TSD) and Healthy Controls (HC)) on the functional connectivity (FC) of the rostral anterior cingulate cortex (rACC) during the supraliminal processing of positive emotional stimuli, in contrast with the processing of emotionally neutral stimuli. Group differences were tested using one-way ANOVA followed by Bonferroni-corrected post-hoc t-tests.

Functional connectivity differences among the three groups were significant in the context of processing happy versus neutral faces. Specifically, in the whole-brain analysis, a significant difference was observed for connectivity of the rACC with the right hippocampus (*p* = 0.036, FDR corrected, see Fig. 1). Post-hoc cluster analyses performed based on extracted beta connectivity values from the significant rACC-hippocampus connectivity found significant differences between the TRD relative to both TSD and HC groups. TRD patients had reduced connectivity compared to TSD (t = −5.24, *p* = 0.000) and HC (t = −3.47, *p* = 0.001) (see Table 2). There were no significant differences in connectivity between TSD and HC (*p* = 0.061) (see Table 2). Table 2 presents the results of the whole-brain analysis. Only clusters that survived FDR correction are reported; no additional clusters reached significance.

**Table 2:** Whole-brain seed-based functional connectivity of the rostral anterior cingulate cortex during supraliminal and subliminal emotion processing tasks.

Task	Contrast	Brain region (aal)	P (FDR corrected)	Cluster size (k)	Peak voxel coordinates (x y z)	Post-hoc
Supraliminal task	Positive > Neutral	Hippocampus	0.036	98	40 −24 −12	HC, TSD > TRD
	Negative > Neutral	-	-	-	-	-
Subliminal task	Positive > Neutral	Cerebellum	0.001	106	−50 −46 −50	TRD > HC > TSD
		Middle temporal gyrus	0.001	99	−36 12 −42	TRD > HC > TSD
		Temporal fusiform cortex	0.04	54	40 −40 −24	HC > TRD,TSD
	Negative > Neutral	Dorsal anterior cingulate cortex	<0.001	80	06 24 24	n.s. (averaged) Disgust: TRD, TSD > HC

There were no significant differences in the FC whole-brain analyses of the rACC for the supraliminal processing of negative emotions versus neutral faces.

#### Subliminal processing

**Fig. 2:**
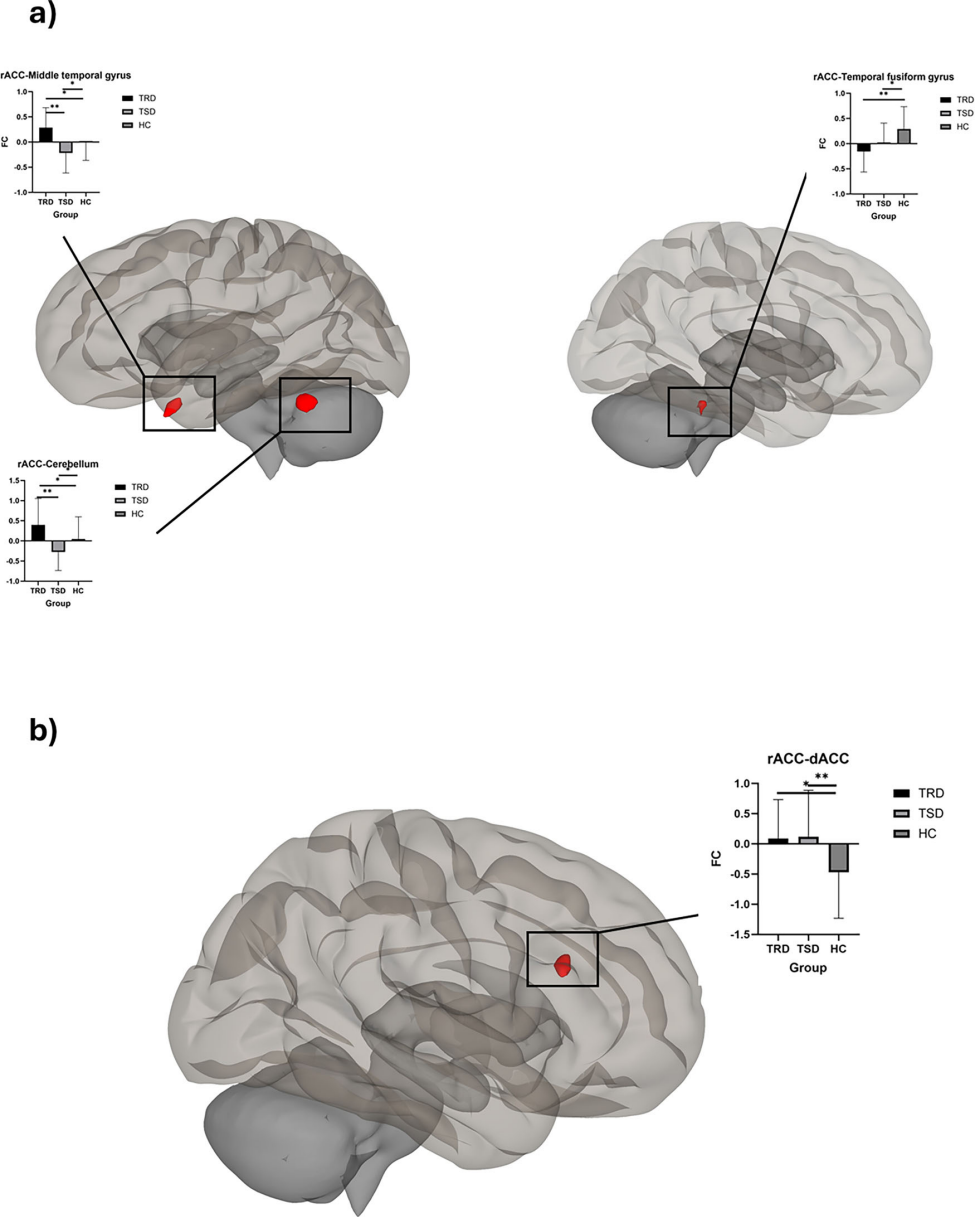
Differences between the groups for the rACC functional connectivity during subliminal processing of emotional stimuli. This illustration offers a visualization of the cluster that contribute to differences between the three groups (Treatment-Resistant Depression (TRD) and Treatment-Sensitive Depression (TSD) and Healthy Controls (HC)) on the functional connectivity (FC) of the rostral anterior cingulate cortex (rACC) during the subliminal processing of emotional stimuli, in contrast with the processing of emotionally neutral stimuli. **a** represents the clusters showing differences between the three groups during the processing of positive versus neutral emotional stimuli, and **b** represents the cluster showing differences between the three groups during the processing of negative versus neutral emotional stimuli. Group differences were tested using one-way ANOVA followed by Bonferroni-corrected post-hoc t-tests.

Significant differences between the three groups in the functional connectivity of the rACC for the subliminal task were found for the processing of happy versus neutral faces. Significant differences were identified for connectivity of the rACC and three clusters in the brain: the left cerebellum (*p* = 0.001, FDR corrected), the left middle temporal gyrus (*p* = 0.001, FDR corrected), and the right temporal fusiform gyrus (*p* = 0.04, FDR corrected) (see Table 2, Fig. 2).

Post-hoc tests revealed significant differences between all three groups for the connectivity between rACC and the cerebellum and between the rACC and the middle temporal gyrus. TRDs had hyperconnectivity compared to TSD and HC groups for both the rACC-cerebellum connectivity (TRD > TSD: t = 4.816, *p* < 0.001; TRD > HC: t = 2.415, *p* = 0.018) and the rACC-middle temporal gyrus connectivity (TRD > TSD: t = 5.154, *p* < 0.001; TRD > HC: t = 3.260, *p* = 0.002). TSD on the other hand had lower connectivity compared to HC for the left cerebellum cluster (t = −2.610, *p* = 0.011) and the left middle temporal gyrus cluster (t = −2.338, 0.022).

For the third cluster i.e. connectivity between the rACC and the right temporal fusiform gyrus, both TRD and TSD groups had lower connectivity than HC but were not different from each other (see Table 2).

For negative versus neutral emotional faces, there was a significant group effect for connectivity between the rACC and the right dorsal ACC. Posthoc analyses revealed that group differences were significant only during the processing of disgust with both the TRD and TSD groups with greater connectivity compared to healthy controls (*p* < 0.001, FDR corrected) but not different from each other (See Table 2).

Due to known biases of neutral facial cues to be interpreted as negative due to psychopathology, we also conducted all analyses contrasting each emotion and implicit baseline i.e. rest presented in Supplementary Material. We also evaluated group differences in connectivity beyond the rACC for other key neural regions involved in emotion processing (amygdala, hippocampus, insula, pregenual and subgenual ACC) in supplementary. Briefly, no significant group differences emerged for the supraliminal tasks. However, during subliminal processing, TRD participants showed altered rACC–frontal orbital cortex connectivity compared to rest, and reduced connectivity between the right hippocampus and sgACC compared to healthy controls during positive emotion processing.

### Clinical factors

There were no significant correlations between the clinical variables and the functional connectivity measures.

## Discussion

This study aimed to elucidate the role of the rostral anterior cingulate cortex in the neural underpinnings of emotion processing in TRD. We wanted to evaluate connectivity related to this region during emotion processing, extending from existing knowledge on altered functional connectivity of the rACC – a key region of the default mode network - in TRD during rest [13].

The rACC is a pivotal node of the DMN, a large-scale brain network extensively studied in major depressive disorder and TRD. Previous neuroimaging studies have shown altered DMN connectivity in TRD, characterized by hyperconnectivity within the DMN and disrupted connectivity between the DMN and other neural networks. Prentice et al. [28] showed that rACC-theta could be an index of treatment resistance across multiple treatments [28].

Furthermore, neuroimaging studies have revealed that the perception of emotion in faces is a complex process, engaging a network of brain regions working together dynamically across time and space, rather than being confined to isolated neural activities in singular areas [38]. These studies highlight the involvement of both supraliminal and subliminal pathways in emotional processing, where supraliminal processing involves the conscious perception of emotions, and subliminal processing occurs without conscious awareness, both of which play crucial roles in how we perceive and respond to emotional facial expressions [39].

The processing of positive and negative emotional faces in the brain engages overlapping yet distinct neural circuits, reflecting the nuanced ways our brains interpret and respond to different emotional stimuli [40]. Understanding these differences is crucial for insights into various psychological conditions. For example, positive emotional faces such as happy facial expressions engage the brain’s reward system [41]. This system is less activated by negative emotional faces, which instead may activate brain areas associated with threat detection and stress response [41]. The rACC, a crucial region for emotional regulation and cognitive processing, appears to exhibit atypical activity suppression during tasks, which Leonards and collaborators [17] associated with disturbances in adaptive neural communication and the dynamic balance between internal and external cognitive modes. These disruptions may form the basis of the maladaptive cognitions and biased emotional processing characteristic of depression [17]. This region is involved in emotion processing and regulation for both positive and negative emotions. However, its engagement can vary depending on the complexity of the emotion processing task and the specific emotional context [42].

In our study, we investigated the functional connectivity underpinnings of the processing of positive and negative emotional expressions, contrasting with the processing of emotionally neutral faces. Additionally, we used both a supraliminal emotion perception task and a subliminal emotion perception task to allow us to measure both explicit and implicit level of emotion processing [7].

For negative versus neutral emotional faces, our analyses revealed a significant group effect only for the supraliminal task and this was for connectivity between the rACC and the dorsal ACC. Posthoc analyses revealed that group differences were significant only during the processing of disgust facial expressions. Both the TRD and TSD groups exhibited greater connectivity between the rACC and the dorsal anterior cingulate cortex compared to healthy controls but not different from each other which suggests a shared neural mechanism underlying the processing of negative emotions, particularly disgust, in both TRD and TSD. The enhanced connectivity between the rACC and dACC might indicate an increased demand for cognitive control and emotional regulation in these groups, reflecting the heightened negative emotional reactivity that is a hallmark of treatment-resistant and treatment-sensitive depression [43]. The lack of significant difference between TRD and TSD groups suggests that this connectivity alteration might be a trait feature in depressive disorders and irrespective of response to treatment.

In contrast for positive vs neutral emotional faces, we saw group differences for both the supraliminal and subliminal processing tasks. For the supraliminal processing task, our analysis revealed a pattern of hypoconnectivity between the rACC and the hippocampus when individuals with TRD processed happy emotions compared to neutral faces, that distinguished them from both the TSD and healthy control groups. This observation aligns with existing literature highlighting hypoconnectivity in reaction to emotional faces in patients with major depression who were non-responsive to antidepressant medication treatments [9]. The observed hypoconnectivity in TRD when processing happy relative to neutral faces could reflect a diminished capacity to modulate neural responses to positive stimuli, a characteristic that might contribute to the anhedonia often seen in TRD, as an expression of impaired positive affect regulation [44]. Existing functional neuroimaging literature corroborates these valence-specific alternations in emotion processing in TRD. Patients with TRD compared with healthy volunteers showed reduced responses to positive emotion within the caudate and insula [7].

Anhedonia, or the diminished ability to experience pleasure, is a core feature of major depressive disorder, and it is particularly pronounced in TRD [44]. The rACC has been implicated in the regulation of emotions and in modulating responses to reward-related stimuli. The link between such neural connectivity patterns and anhedonia is supported by findings that suggest alterations in reward processing circuits are associated with anhedonia in depression [44]. Specifically, reduced activation in the ventral striatum (VS), an area closely connected to the rACC and involved in reward processing, has been correlated with the severity of anhedonia in MDD [44]. Ang et al. [45] demonstrated that higher resting-state connectivity between the nucleus accumbens and rACC predicted response to bupropion, but not sertraline, after antidepressant medication nonresponse [45]. This suggests VS-rACC connectivity may help identify patients likely to benefit from dopaminergic antidepressants. However, we did not observe connectivity differences between the rACC and the ventral striatum for our tasks which might suggest this neural mechanism is better evident in tasks that directly tap reward processing functions.

The connectivity of the rACC with the hippocampus, an area which is crucial for memory formation and retrieval, suggests a pathway through which emotional experiences are integrated and stored. This connectivity is believed to play a role in how emotional memories influence current emotional states and decision-making [46]. The hippocampus is involved in the cognitive processing of emotional memory, so the decreased functional connectivity between the rACC and hippocampus may be related to abnormal regulation of emotional memory in TRD [47]. Interestingly, our findings did not indicate significant differences in the FC of the rACC with the hippocampus between TSD and HC, suggesting that this altered connectivity might be specific to TRD. Additionally, literature on the connectivity between the DMN and hippocampus indicates hyperconnectivity in MDD patients [9]. In our study, we not only found that this hyperconnectivity is present in patients who are currently depressed (the TRD group), but it is not present in the TSD group (patients with depression who recovered), highlighting a unique connectivity pattern in TRD.

Previous literature also has shown increased hippocampal response to positive stimuli associated with treatment response [48], suggesting that high levels of activity in the hippocampal region at baseline may indicate a resilience in neural responsivity in those patients who subsequently showed a clinical improvement [48]. Therefore, hypoconnectivity between the rACC and the hippocampus, a region involved in memory and emotion processing [49, 50], may signal a disrupted neural circuit that contributes to the diminished sensitivity to positive stimuli seen in TRD. This disruption could undermine the ability of individuals with TRD to engage with and retain positive emotional experiences, further perpetuating the cycle of depression.

One of the most notable findings on the subliminal task was the hyperconnectivity between the rACC and the cerebellum also during happy face processing, where connectivity was greatest in TRD compared to HC and TSD. This result is particularly interesting as it introduces a novel aspect of cerebellar involvement in TRD. Traditionally associated with motor function and coordination, the cerebellum has increasingly been recognized for its role in cognitive and emotional processing [51]. The involvement of the cerebellum in emotion processing alterations aligns with prior evidence linking this region to affective regulation across psychiatric disorders. For example, Turner et al. [52] showed cerebellar activation in response to happiness-evoking stimuli [52], while more recent work by Siciliano et al. [53] implicated the cerebellum in predicting social outcomes in bipolar disorder [53]. These findings reinforce the view of the cerebellum as a transdiagnostic node in affective and social processing networks. The heightened connectivity observed in TRD in this study could suggest an adaptive or maladaptive mechanism where the cerebellum compensates or exacerbates emotional regulation difficulties, particularly in the non-conscious processing of positive emotions. This finding prompts further investigation into how the DMN might interact abnormally with the cerebellum in TRD, potentially disrupting normal emotional regulation.

Additionally, we also observed hyperconnectivity of the rACC with the middle temporal gyrus in the subliminal processing of positive emotions, that distinguished TRD from both HC and TSD. While previous literature commonly associates major depression with decreased activity in the middle temporal gyrus [54], the observed rACC hyperconnectivity to this region in TRD suggests a distinct neural pattern that differentiates it from TSD. The middle temporal gyrus is implicated in the semantic processing of emotions and social cognition [55]. The increased connectivity could reflect an over-engagement or faulty modulation of this region when processing happiness subliminally, pointing to a possible neural basis for the altered perception and integration of positive social cues in TRD.

The third significant finding during the subliminal processing of positive emotions involved the right temporal fusiform gyrus, where HC exhibited greater connectivity than both TRD and TSD. This aligns with existing research indicating reduced fusiform gyrus activity in depression during the processing of facial emotions [54]. The fusiform gyrus plays a crucial role in face recognition and the interpretation of facial expressions. Its reduced involvement in both TRD and TSD groups suggests a shared impairment in processing emotional faces at a non-conscious level, potentially contributing to the social cognition deficits observed in depressive disorders in general rather than specifically to TRD.

The distinct patterns of connectivity observed during supra versus subliminal processing tasks imply a disconnection in how emotions are processed at different levels of awareness in TRD. Non-conscious processing tasks revealed significant connectivity between the rACC and areas typically associated with automatic emotional responses, such as the cerebellum and middle temporal gyrus. This suggests that individuals with TRD may have heightened automatic emotional reactivity, particularly in response to positive stimuli, which is not accessible to conscious awareness and could contribute to the persistent negative emotional bias seen in this disorder. In contrast, supraliminal emotion processing did not show these patterns, indicating that the rACC plays a role in both levels of processing in TRD but engages different regions. Specifically, during subliminal tasks, the rACC connects with automatic processing regions, while during conscious tasks, this region has altered hyperconnectivity with the hippocampus, which is involved in deliberate emotional processing. The lack of observed differences in automatic processing regions during the supraliminal task could be due to the fact that the task is not sensitive enough to capture these mechanisms, rather than these processes being normal in TRD. The differences seen in the subliminal task highlight the complexity of emotional processing in TRD. Collectively these findings underscore a complex network dysfunction in TRD, where the rACC’s impaired modulation of neural responses to positive stimuli and its altered connectivity with key regions of the emotion processing network, namely the hippocampus and the middle temporal gyrus.

We acknowledge several limitations in this study. First, the cross-sectional design constrains our ability to infer causality between the observed alterations in functional connectivity and treatment resistance. To better understand the dynamic changes in connectivity and their relationship with treatment outcomes, future research should adopt longitudinal designs. This would also help untangle trait vs state markers of treatment resistance.

Secondly, although we collected detailed information on current medication use, the small number of participants taking the different classes of medications limited our ability to control for these variables in the main analyses. While such medications may influence functional connectivity, their low frequency in the sample precluded meaningful covariate adjustment and should be considered when interpreting the findings.

Additionally, despite careful matching, our sample size is relatively modest, urging caution in the extrapolation of our findings. Studies with larger and more diverse cohorts are essential for corroborating our results. This study’s focus on emotion processing through facial expression tasks also presents a limitation. Face processing is a multifaceted function, engaging a network of brain regions often termed the “core face network,” including the fusiform face area, occipital face area [56], and superior temporal sulcus [40]. Not all relevant regions within this network were examined in our analysis as our focus was primarily on emotion processing. Future research should distinguish between functional connectivity in face processing and broader emotion processing by employing diverse emotional processing paradigms. The same applies for emotion processing versus emotion regulation.

The sample’s heterogeneity is another notable limitation, along with the study’s focus on HAMD-21 symptoms, excluding a thorough investigation of anhedonia. This aspect is critical for a clinically meaningful interpretation of our findings especially considering that differences were for processing of happy emotions. While anhedonia is particularly relevant in TRD, our ability to explore its neural correlates was limited in this study. Although the HAMD was administered, all TRD participants endorsed anhedonia on the relevant items, resulting in no variability within the group. This precluded meaningful sub-analyses based on anhedonia severity. Future studies should incorporate dimensional, self-report measures that more sensitively capture variation in anhedonia to better investigate its association with neural function.

In conclusion, our findings, along with the broader literature, suggest a critical role for the rACC in the disrupted emotional processing observed in TRD. The abnormal connectivity patterns between the rACC and regions such as the hippocampus and middle temporal gyrus may reflect disruptions in circuits that support emotion regulation and memory integration—functions that are relevant to depressive symptomatology and treatment responsiveness. The findings suggest that TRD involves distinct neural pathways for processing emotional stimuli under conscious and non-conscious conditions. Specifically, non-conscious processing seems to involve heightened connectivity between the rACC and brain regions like the cerebellum and the middle temporal gyrus in TRD compared to TSD and controls. This may indicate that while these regions are more responsive or exhibit altered connectivity in TRD, suggesting that changes in connectivity can be elicited during unconscious emotional processing, without the need for conscious awareness. Such disruptions in automatic emotional processing could contribute to persistent symptoms, such as pervasive sadness or anhedonia, despite treatment. While the rACC appears to be engaged during non-conscious emotional tasks, its reduced connectivity with regions like the hippocampus may reflect difficulties in integrating these automatic responses into broader regulatory processes. This pattern could indicate a disconnect between early affective processing and higher-order emotional regulation, though further research is needed to directly assess these functional implications.

Addressing these neural circuitry disruptions holds promise for advancing our understanding of the role of the rACC in positive emotion processing in TRD, paving the way for interventions that more precisely target the neural bases of the disorder’s core symptoms.

## Supplementary information

Supplementary Material

## Data Availability

The data that support the findings of this study are available from the corresponding author upon reasonable request.
