# Supplementary material for: Role of the rostral anterior cingulate cortex in emotion processing in Treatment Resistant Depression
Source: Transl Psychiatry. 2025 Oct 6;15:378. doi: 10.1038/s41398-025-03600-3 (PMC12500956; doi:10.1038/s41398-025-03600-3)
Supplement: Supplementary file 1 — Supplementary Material [file 41398_2025_3600_MOESM1_ESM.docx]

**Supplementary section**

**Supplementary Methods**

*ROI to ROI analyses – Emotion Processing Network:*

While the seed-to-voxel analyses were conducted to investigate whole-brain connectivity patterns emanating from the rACC, exploratory ROI-to-ROI analyses were also conducted. The aim of theses secondary analyses is to investigate how the connectivity of other regions of the emotion processing network [1], beyond the rACC, differentiates between the groups. The emotion processing network is involved in the measurement of the emotional tone of the facial expression, and includes the amygdala, the insula, the hippocampus, and the subregions of the anterior cingulate cortex [2]. These regions do not operate in isolation but are part of a dynamic network that processes emotional stimuli, integrating sensory, cognitive, and memory-related aspects to generate appropriate emotional and behavioral responses [2].

This dual approach allowed us to construct detailed connectivity maps and matrices, providing a nuanced understanding of the interactions between these critical brain regions in each subject under positive and negative emotion processing.

The regions of the emotion processing network used in the ROI to ROI analyses are: bilateral amygdala, bilateral insula, bilateral hippocampus, rACC and sgACC. The ROI masks for the right and left amygdala, right and left hippocampus, right and left insula regions were extracted from the Gordon atlas [3]. The selection of sgACC ROI anatomic landmark for Brodmann Area 25 (BA25) [4]. The ROI for BA25 was generated from the AAL atlas [5]. The rACC ROI was the same as the one used for the seed-based analyses.

For the ROI to ROI analyses, an analyses-level corrected p < 0.05 threshold was considered (to control the likelihood of making any Type I errors across the entire set of pre-defined ROI comparisons), as the connectivity is assessed between pairs of predefined ROIs rather than multiple voxels.

*Whole brain seed-based functional connectivity analyses – Emotion vs Rest:*

We performed exploratory functional connectivity analyses that complement the main findings described in the manuscript. These analyses were conducted to explore additional connectivity patterns associated with the rostral anterior cingulate cortex (rACC) and the affective network, contrasting specific emotional states and the brain at rest.

Investigating the contrast between specific emotional states and a baseline resting state provides critical insights into the neural dynamics underlying emotional experiences. This approach is particularly pertinent given the propensity for individuals with treatment-resistant depression (TRD) to exhibit a negative processing bias, wherein even neutral stimuli can be interpreted negatively [6]. This bias underscores the importance of contrasting emotional conditions with rest to elucidate the distinct neural activations associated with emotional processing and to differentiate them from the brain's baseline activity. Given their exploratory nature, the results should be interpreted as preliminary, guiding future hypothesis-driven research to further elucidate the role of these connectivity patterns in emotional processing and mood dysregulation.

**Supplementary Results**

*Whole-brain seed-based functional connectivity analyses – Emotion vs Rest:*

Supraliminal processing:

No significant differences between the groups were identified for the functional connectivity of the rACC during this task, neither for the processing of positive nor negative emotions.

Subliminal processing:

The whole-brain analysis indicated significant differences in the functional connectivity of the rACC with the bilateral frontal orbital cortex during the processing of positive emotion, when comparing with resting-state (see Supplementary Table 1). Post hoc tests revealed a pattern of hyperconnectivity in the TRD group for the right frontal orbital cortex during the processing of positive emotions, and a pattern of hypoconnectivity in the TRD group for the left frontal orbital cortex during the processing of negative emotions. Reversely, a pattern of hyperconnectivity in TRD was found for the left frontal orbital cortex during the processing of negative emotions, and a pattern of hypoconnectivity in the TRD group for the right frontal orbital cortex during the processing of positive emotions.

*ROI to ROI – Emotion processing network*

Supraliminal processing:

No significant differences in the functional connectivity within the affective network emerged for the processing of happy versus neutral faces, or the processing of negative emotions versus neutral faces for the supraliminal task.

Subliminal processing:

Significant differences in the functional connectivity of the right hippocampus with the sgACC were found during the processing of positive emotions (versus neutral faces), specifically between the TRD and the HC groups (p=0.03, FDR corrected; HC>TRD).

No significant differences were found for the functional connectivity for Negative vs neutral condition within the affective network during the subliminal task.

*ROI to ROI analyses – Emotion vs Rest:*

No significant differences between the groups were founds for the functional connectivity of the affective network, for any of the tasks or contrasts, when contrasted with rest.

**Discussion**

Additionally, our ROI-to-ROI analyses during subliminal emotion processing revealed hypoconnectivity between the sgACC and the right hippocampus in TRD, consistent with the hypoconnectivity observed between the rACC and the hippocampus during supraliminal emotion processing. This overlap emphasizes the disrupted connectivity involving the hippocampus within the affective network in individuals with treatment-resistant depression, specific to positive emotion processing, even during subliminal level of emotion processing.

**Supplementary Tables**

**Supplementary Table 1.** Whole brain functional connectivity of the rACC compared between the three groups, for the supraliminal and subliminal processing of positive and negative emotions contrasting with rest.

| Task | Contrast | Brain region (aal) | P (FDR corrected) | Cluster size (k) | Peak voxel coordinates (x y z) | Post-hoc |
| --- | --- | --- | --- | --- | --- | --- |
| Faces Supraliminal | Positive vs Rest | - | - | - | - | - |
|  | Negative vs Rest | - | - | - | - | - |
| Faces Subliminal | Positive vs Rest | Frontal orbital cortex L | 0.009 | 59 | -46 32 -14 | HC, TSD > TRD |
|  |  | Frontal orbital cortex R | 0.026 | 41 | 18 12 -28 | TRD > HC, TSD |
|  | Negative vs Rest | Frontal orbital cortex R | <0.001 | 100 | 18 12 -28 | HC, TSD > TRD |
|  |  | Frontal orbital cortex L | 0.01 | 63 | -46 32 -14 | TRD > HC, TSD |

Supplementary table 2

| **Clinical variable** | **FC variable** | **Correlation (r)** | **Significance (p)** | **Notes** |  |
| --- | --- | --- | --- | --- | --- |
| Age | C_HvN | -0.077 | 0.442 | Not significant |  |
|  | NC_HvN1 | 0.151 | 0.128 | Not significant |  |
|  | NC_HvN2 | 0.091 | 0.362 | Not significant |  |
|  | NC_HvN3 | -0.034 | 0.733 | Not significant |  |
|  | NC_NvN_Disgust | -0.100 | 0.314 | Not significant |  |
|  | NC_PvR1 | 0.041 | 0.682 | Not significant |  |
|  | NC_PvR2 | -0.010 | 0.921 | Not significant |  |
|  | NC_NvR1 | 0.011 | 0.913 | Not significant |  |
|  | NC_NvR2 | -0.007 | 0.942 | Not significant |  |
| No. of previous episodes | NC_HvN1 | 0.092 | 0.549 | Not significant |  |
|  | NC_HvN2 | 0.417 | 0.244 | Not significant |  |
|  | NC_HvN3 | -0.178 | 0.244 | Not significant |  |
| No. of prev. episodes | | NC_NvN_Disgust | 0.120 | 0.432 | Not significant |
|  |  | NC_PvR1 | -0.043 | 0.780 | Not significant |
|  |  | NC_PvR2 | -0.052 | 0.736 | Not significant |
|  |  | NC_NvR1 | -0.115 | 0.453 | Not significant |
|  |  | NC_NvR2 | -0.083 | 0.587 | Not significant |
| Age at first episode | NC_HvN1 | 0.051 | 0.689 | Not significant |  |
|  | NC_HvN2 | -0.061 | 0.636 | Not significant |  |
|  | NC_HvN3 | 0.011 | 0.930 | Not significant |  |
| Length of time on ADM | NC_HvN1 | 0.239 | 0.086 | Not significant |  |
|  | NC_HvN2 | 0.224 | 0.407 | Not significant |  |
|  | NC_HvN3 | -0.014 | 0.920 | Not significant |  |
|  | NC_NvN_Disgust | 0.019 | 0.895 | Not significant |  |
|  | NC_PvR1 | -0.182 | 0.192 | Not significant |  |
|  | NC_PvR2 | 0.162 | 0.247 | Not significant |  |
|  | NC_NvR1 | -0.223 | 0.108 | Not significant |  |
|  | NC_NvR2 | 0.201 | 0.149 | Not significant |  |

Correlation is significant at the 0.01 level (2-tailed).

**Supplementary references:**

1. Korgaonkar, M. S., Erlinger, M., Breukelaar, I. A., et al. (2019). Amygdala activation and connectivity to emotional processing distinguishes asymptomatic patients with bipolar disorders and unipolar depression. *Biological Psychiatry: Cognitive Neuroscience and Neuroimaging, 4*(4), 361-370.
2. Adolphs, R. (2002). Neural systems for recognizing emotion. *Current Opinion in Neurobiology, 12*(2), 169-177.
3. Gordon, E. M., Laumann, T. O., Adeyemo, B., Huckins, J. F., Kelley, W. M., & Petersen, S. E. (2016). Generation and evaluation of a cortical area parcellation from resting-state correlations. *Cerebral Cortex, 26*(1), 288-303. doi: 10.1093/cercor/bhu239.
4. Brodmann, K. (2005). *Brodmann’s: Localisation in the cerebral cortex* (L. J. Carey, Trans.). Berlin: Springer.
5. Tzourio-Mazoyer, N., Landeau, B., Papathanassiou, D., Crivello, F., Etard, O., Delcroix, N., Mazoyer, B., & Joliot, M. (2002). Automated anatomical labeling of activations in SPM using a macroscopic anatomical parcellation of the MNI MRI single-subject brain. *NeuroImage, 15*(1), 273-289. doi: 10.1006/nimg.2001.0978.
6. Gotlib, I. H., & Joormann, J. (2010). Cognition and depression: current status and future directions. *Annual Review of Clinical Psychology, 6*, 285-312.
